# Recurrent haemorrhagic ovarian cyst and anticoagulant therapy: a case report with review of treatment modalities

**DOI:** 10.11604/pamj.2021.40.52.30961

**Published:** 2021-09-21

**Authors:** Kavitha Nagandla, Mohamed Faiz Bin Mohamed Jamli, Faridah Hanim, Joanne Lim Xu Mei, Siti Fathima Syazliana Din

**Affiliations:** 1 Department of Obstetrics and Gynaecology, International Medical University, Clinical Campus, Jalan Rasah, 70300 Seremban, Negeri Sembilan, Malaysia,; 2 Department of Obstetrics and Gynaecology, Hospital Tuanku Jaffar, Jalan Rasah, 70300 Seremban, Negeri Sembilan, Malaysia

**Keywords:** Haemorrhagic, ovarian cyst, anticoagulants, management, surgery

## Abstract

The common gynaecological causes of acute pelvic pain include ruptured ectopic pregnancy, haemorrhagic corpus luteal cyst or torsion of an ovarian cyst. Ovarian vascular accidents are reported in women on oral anticoagulation presenting as an acute pelvic pain. Although such vascular accidents with anticoagulation therapy are an unusual entity, a meticulous history, clinical examination, and laboratory workup to confirm the diagnosis and timely intervention is needed to reduce attending morbidity and mortality. However, a standard algorithm for management is not described in the literature. We hereby report successful management of recurrent hemorrhagic ovarian cyst due to coagulopathy in a woman with mechanical heart valves with timely surgical intervention. This case report discusses operative versus non operative management approach and may provide value addition to readers encountering such cases in their clinical practice.

## Introduction

Women with mechanical prosthetic heart valves are considered high risk population with increased incidence of thromboembolic events [1]. Therapeutic anticoagulation as long-term measure is effective in preventing future thromboembolic episodes and their associated mortality [2]. Spontaneous bleeding is one of the most common adverse effects of warfarin, and factors such as old age, dose, duration of therapy, drug interaction and occult diseases further determine the risk of bleeding [3]. Strict monitoring of the coagulation profile is therefore paramount in patients taking long-term anticoagulation. Major gynecological bleeding complications from anticoagulation therapy although are rare, cases have been reported of hemorrhagic rupture of luteal cysts in premenopausal women in the absence of concurrent ovulation suppression [4]. The reported incidence of hemorrhagic ovarian cysts is 1% among women receiving anticoagulation medications and frequently a complication when International Normalized Ratio (INR) is more than 4 [5]. Life-threatening bleeding can occur with cystic hemorrhage from rupture, with an associated mortality rate from resultant hemoperitoneum of more than 11% [6]. Such patients present a challenge, as abrupt reversal of anticoagulation is required to control any ongoing hemorrhage and further surgical blood loss [7]. Currently, there are no clear recommendations regarding the modalities required to reverse anticoagulation for emergency surgery and the ideal time to restart anticoagulation therapy safely [7]. We describe a case of recurrent hemorrhagic ovarian cyst in premenopausal women with mechanical heart valves on anticoagulation therapy who presented in emergency with acute abdomen and was managed successfully with emergency exploratory laparotomy and adequate blood products.

## Patient and observation

**Patient information:** a 47-year-old nulliparous female presented to an emergency department with left lower abdominal pain for the last three days. She has an underlying history of chronic rheumatic heart disease and mitral valve replacement and is anticoagulant therapy with warfarin 2.5 mg once daily since 1994. She has underlying history of hypertension and diabetes mellitus which has been well controlled. She has a history of emergency laparotomy for haemorrhagic ovarian cyst in 2005 and cystectomy and bilateral tubal ligation performed as she was not keen for fertility in view of her underlying cardiac condition. Furthermore, she is diagnosed with bilateral endometrioma since 2018 and is on conservative management. Her last menstrual period was twenty days ago, with regular menstrual history and moderate flow.

**Clinical findings:** on examination, she was pale, and there is presence of diffuse tenderness on per abdominal examination. The cardiovascular examination revealed mechanical valve click, pulse rate of 108beats/minute blood pressure of 100/60 mm Hg. Per speculum revealed healthy cervix and on vaginal examination: uterus- normal-sized, anteverted, bilateral fornix-fullness present.

**Diagnostic assessment:** on initial laboratory work-up, she was noted to have a hemoglobin (Hb) of 10g/dL, hematocrit of 33.6%, and platelet count of 248, INR of 2.7 with normal prothrombin time and activated partial thromboplastin time. Urine pregnancy test was negative. An ultrasound scan of the pelvis revealed an adnexal lesion with mixed echogenic adnexal mass abutting the uterus. There is no free fluid seen. She developed a spike of fever on the second day of admission and septic workup was initiated. Her total white blood cells increased from 13.3 to 16.1cells/mm^3^.

**Figure 1:**
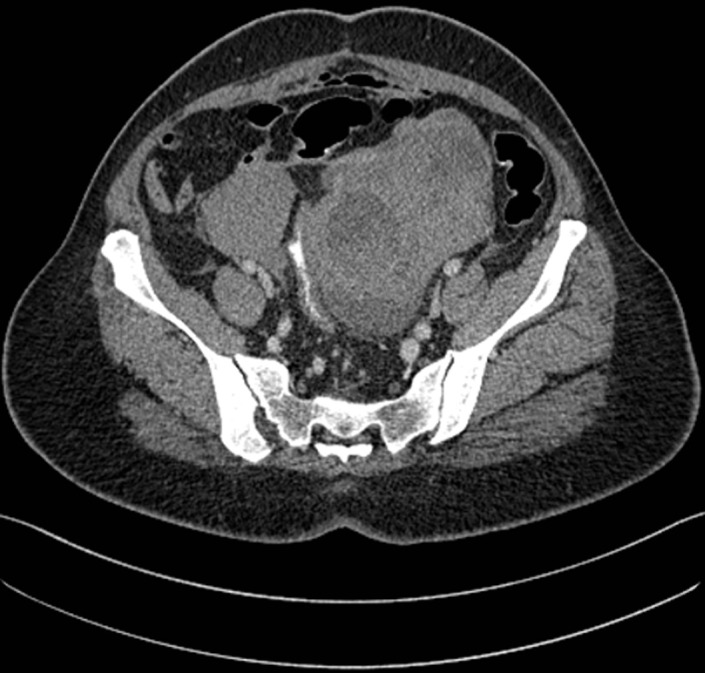
computerized tomography (CT) of pelvis showing bilateral enlarged irregular heterogeneously enhancing masses; several cystic components seen in the mass suggestive of necrosis; no contrast extravasation suggestive of active bleeding is seen

**Diagnosis:** a provisional diagnosis of infected endometrioma was made, and she was started on Intravenous cephalosporins. Her COVID polymerase chain reaction (PCR) was negative. An echocardiography showed normal functioning valves. She was afebrile 24 hours post antibiotic administration and a decision was made to manage her conservatively. On Day 4 of admission, her hemoglobin dropped to 6 g/dl. A computerized Tomography of the pelvis revealed large bilateral adnexal masses with no active extravasation, indicating no active bleeding (Figure 1). However, INR was 6.6 and cardiology decision was to stop warfarin due to overanticoagulation related to concomitant drug interaction with cephalosporins and possible risk of ongoing hemorrhage.

**Figure 2:**
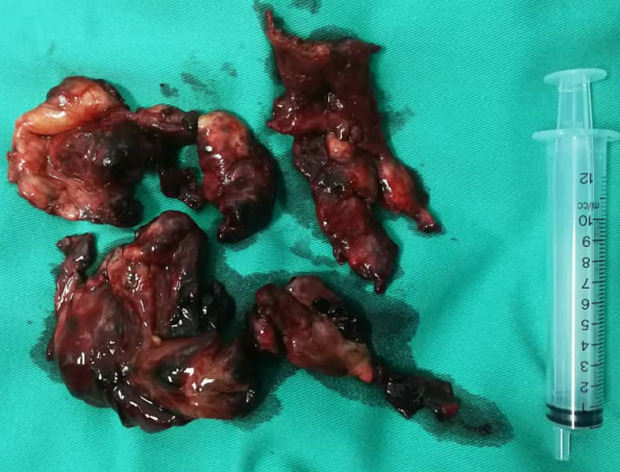
specimen of bilateral hemorrhagic ovarian cysts that ruptured that on intraoperative manipulation with 200-300cc clots

**Therapeutic interventions:** in view of acute anemia, she was planned for emergency laparotomy and total abdominal hysterectomy with bilateral salpingo-oophorectomy was proceeded as patient requested for a definitive management due to underlying cardiac condition. Preoperatively she was transfused with 2 units of whole blood and Intravenous vitamin K 2 mg for warfarin reversal. Intraoperative findings include bilateral adnexal masses, that ruptured on manipulation with 200-300cc clots from both the ovaries (Figure 2). During adhesiolysis of dense adhesions related to her previous surgery, there was an iatrogenic bladder and serosal tear of sigmoid colon and repair was performed respectively.

**Follow-up and outcome of interventions:** postoperatively, she was transfused with 2 units whole blood and monitored with daily INR. Her hemoglobin improved to 9 g/dl. In view of hematuria immediate postoperative period, and risk of bleeding, a decision was made to delay the initiation of anticoagulants. Her INR was 1.8-2.2 and she was started on subcutaneous low molecular weight heparin (LMWH) until 10^th^ post-operative day and bridged with warfarin 2.5 mg following normalization of INR for at least 48 hours. She was discharged uneventfully on 14^th^ post-operative day. Histopathology report was suggestive of haemorrhagic ovarian cyst.

**Patient perspective:** at 6 weeks follow-up in combined Gynaecology and Medical Clinic, she had normal bladder and bowel functions and had expressed satisfaction that a definitive treatment has been achieved. She had given the authors permission to publish this case report.

## Discussion

It is well known that patients with prosthetic heart valves require lifelong anticoagulation to prevent thromboembolic events [2]. However, prolonged anticoagulation presents with future complications such as significant bleeding diathesis that can be life-threatening requiring emergency interventions [3]. Bleeding conditions include gastrointestinal bleed, intracranial bleed, menorrhagia, and ovarian haemorrhage due to rupture of follicle or corpus luteum [2]. There are many published reports as case series of corpus luteal haemorrhage, however there are few reports addressing bleeding in women with mechanical heart valves [8-10]. Our case further adds to the literature. The overall estimated mortality in this group of patients has been reported as 3-11% and recurrence risk of 25-31% of cases [11]. It is paramount to have a close monitoring of coagulation profile in patients on long-term anticoagulants to prevent such complications.

Currently, there is no standard algorithm in the management of patients presenting with haemorrhagic ovarian cysts. Barel *et al*. reported abdominal pain as a prevalent and constant symptom in all patients; 10.7% also had fever, 13% had nausea and vomiting, and 4% showed urinary disorders [12]. It is worth mentioning that it is not always clinically possible to differentiate hemorrhagic cyst and ruptured hemorrhagic cyst. Although several previous studies have reported a rate of surgery for ruptured hemorrhagic cysts with hemoperitoneum as high as 80% [13]. Conservative management is usually performed with analgesia and close observation in actual clinical settings, which is supported by recent advances in imaging studies that allow an early and accurate diagnosis [14]. In a recent study, the role of pre-treatment computed tomography (CT) that can predict the necessity of surgical treatment based on image findings were reported. It is identified that presence of active bleeding and depth of hemoperitoneum on axial plane are potential risk factors for surgery in these patients [15].

The principles of treatment are aimed at controlling the bleeding and salvage the ovaries [14]. However, it is not always possible to adopt a conservative approach and surgical approach is restored in the event of hemodynamic instability or diagnostic dilemma similar to our case where the CT findings suggestive of adnexal cyst with no active bleeding [15]. A contrast-enhanced CT of the abdomen is recommended over ultrasound to initiate correct management in a timely fashion [15]. However, few observations in literature have challenged validity of surgical modality as first therapy. First, hemoperitoneum up to 1litre gets absorbed within a week and non-inflammatory ovarian bleeding is not associated with intra-abdominal adhesions [16].

If surgical modality is the option, there is ambiguity regarding the treatment to reverse anticoagulation for emergency surgery and the ideal time to restart anticoagulation therapy safely [7]. The possible options for reversal of anticoagulation include vitamin K, prothrombinase complex concentrate (PCC) and fresh frozen plasma (FFP) [3]. The most immediately available is FFP and vitamin K. It is identified that there is PCC-associated thrombotic risk because of high level of factor II in the PCC (relative to the other factors), which is known to increase thrombin generation especially in the situation of mechanical heart valves [3]. With regard to restarting anticoagulation in patients with warfarin-induced major bleeding and mechanical heart valves, the safe period varies from 7-14 d after the onset of bleeding [8]. As ovarian cyst hemorrhage may reoccur, ovulation suppression should be considered. There is a good evidence for prescribing depot medroxyprogesterone (DMPA) to suppress ovulation in anticoagulated patients with prosthetic heart valves [17]. However, in our case, there was no preceding formal recommendation of hormonal therapy for ovarian suppression.

## Conclusion

Patients who are on oral anticoagulants have more chances of bleeding tendencies and higher risk of recurrences of haemorrhagic ovarian cyst. Massive hemoperitoneum resulting from an ovarian cyst rupture is rare, but potentially life-threatening if not diagnosed and treated emergently. Conservative management is an option in hemodynamically stable patients. However, a low threshold of surgical intervention must be the approach in the presence of diagnostic dilemma with emergency reversal of anticoagulation and restarting therapy postoperatively as guided by the potential risk of rebleeding. Although the risks of specific hormonal contraceptives in this patient population still warrant further investigation, hormonal contraception can be safe and efficacious therapy for ovulation suppression and prevention of future recurrences.
